# Regression of Cervical Cystic Hygroma with a Single Session of Sclerotherapy

**Published:** 2014-05-21

**Authors:** Ghulam Mustafa, Muhammad Saleem

**Affiliations:** Department of Pediatric Surgery The Children’s Hospital and the Institute of Child Health, Lahore

A 1-month-old child presented with cystic hygroma measuring 14 cm x10 cm on the left side of neck. Injection bleomycin was injected into the swelling after aspirating it completely with a wide bore needle. The aspirate was straw colored. The patient was discharged on the next day. At one month follow-up, it was completely regressed with only minor skin pigmentations and redundant skin (Fig 1, 2).

**Figure:**
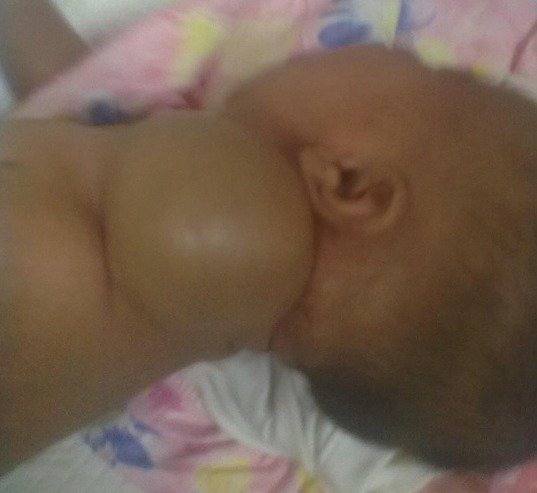
Figure 1: Cystic hygroma

**Figure:**
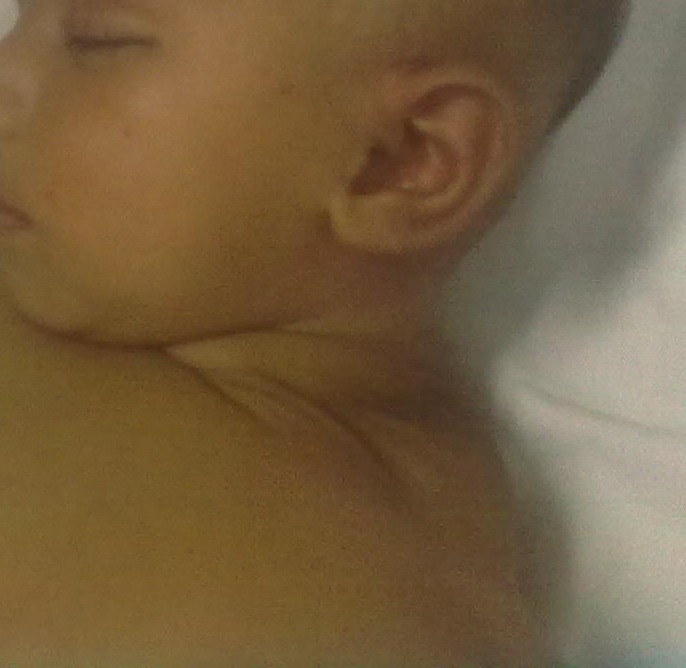
Figure 2: After 1 month of single session sclerotherapy.

## DISCUSSION

Macrocystic lymphangioma which was previously known as cystic hygroma is a congenital malformation of the lymphatic system. Complete surgical excision is preferred but as there is risk of damage to vital structures it is largely superseded by intralesional sclerotherapy.[1-2] Complete resolution of cystic hygroma with injection bleomycin can be achieved in about 60% of cases and requires multiple sclerotherapy sessions. Very rarely, the complete resolution is achieved in a single session as happened in our case.

## Footnotes

**Source of Support:** Nil

**Conflict of Interest:** None declared

